# Bimodal Q-band probehead with improved signal-to-noise ratio in pulse electron paramagnetic resonance

**DOI:** 10.5194/mr-7-21-2026

**Published:** 2026-03-20

**Authors:** Vasyl Denysenkov, Alexey Fedotov, Burkhard Endeward, Thomas F. Prisner

**Affiliations:** 1 Institute for Physical and Theoretical Chemistry, Goethe University 60438 Frankfurt, Germany; 2 A.V. Gaponov-Grekhov Institute of Applied Physics of the Russian Academy of Sciences, Nizhny Novgorod, 603950, Russia

## Abstract

In addition to the development of various resonators, the concept of a probehead equipped with an additional low-noise amplifier (LNA) is becoming increasingly popular to enhance the sensitivity of electron paramagnetic resonance (EPR) spectrometers. The low-noise-detection amplifier makes it possible to measure pulsed EPR signals with high sensitivity. However, a strong reflected pulse signal can cause saturation and deterioration of the LNA characteristics, which requires protection of the LNA (for example, by using a protection switch in front of the LNA), which, in turn, reduces the signal-to-noise ratio. To overcome these limitations, we propose using an EPR probehead based on a bimodal cavity with strong isolation between the input and output ports in combination with a low-noise amplifier connected to the cavity output. The experiments demonstrate a 4-fold increase in the signal-to-noise ratio (SNR) of a bimodal probehead operating in transmission mode compared to its operation in reflection mode, which was achieved thanks to the additional use of LNA. The performance of the probe was also compared with the Bruker EN 5107D2 probe available in our laboratory, which showed an improvement that can be achieved by increasing the SNR by 2 times due to additional LNA and isolation of the detection channel from the input signal and by 3.3 times due to a larger sample volume in the bimodal probe ( ∼ 20 µ L) at Q-band frequencies compared to the Bruker one ( ∼ 6 µ L). The developed probehead can be used together with commercial Bruker ELEXYS EPR spectrometers without modification of the microwave bridge.

## 1 Introduction

Electron paramagnetic resonance (EPR) spectroscopy is a well-established method for studying systems with unpaired electrons. It is widely used in research areas such as chemistry, physics, medicine, biology, and material science. Increasing the sensitivity of EPR spectrometers is important for the development of new methods that open up new application possibilities.

The central component of any conventional EPR spectrometer is a resonator, which amplifies the excitation, as well as the induced microwave (mw) signal, in the sample, thereby determining the sensitivity of measurements. Single-mode cavities (Reijerse et al., 2012) and dielectric resonators (Hyde and Mett, 2017; Raitsimring et al., 2012) are the most commonly used resonators operating in the reflection mode, used in almost all EPR spectrometers. For some specific applications, more sophisticated options such as loop-gap resonators (LGRs) (Hyde and Froncisz, 1989; Rinard and Eaton, 2005; Simovic et al., 2006; Forrer et al., 2008; Tschaggelar et al., 2017), photonic band gap resonators (Milikisiyants et al., 2018), plasmonic metasurface resonators (Tesi et al., 2021), microresonators (Usevicius et al., 2025; Twig et al., 2013), and Fabry–Perot resonators (Tipikin et al., 2010; Neugebauer and Barra, 2010; Budil and Earle, 2004) were developed for measurements in reflection mode depending on the used mw frequency range and the condition of the sample. A further example is a dual-mode cavity that matches both mw excitation frequencies in experiments with pulsed electron–electron double resonance (PELDOR/DEER), which increases the sensitivity of such experiments with a large frequency separation of the mw excitation pulses (Tkach et al., 2011). However, when operating in reflection mode, a significant part of the mw excitation power returns to the mw bridge due to resonator ring-down under pulse EPR conditions and may reduce the sensitivity of the spectrometer receiver due to insertion losses in the protection gate switches in pulse mode and due to the mw source noise in continuous-wave (CW) mode.

A well-known approach to avoid the ringing, as well as to reduce the problem with the source noise, is the use of a bimodal cavity in which two modes with orthogonal H-field polarization resonate at the same frequency, permitting detection of the orthogonal component of the circularly polarized induction signal, i.e., to excite the x component of the magnetization and to detect the y component (Huisjen and Hyde, 1974; Mailer et al., 1980; Barendswaard et al., 1984; Prisner and Dinse, 1989). This approach has also been used with loop-gap resonators (Piasecki et al., 1996) and cross-loop resonators (Rinard et al., 1996), as well as for a non-resonant probehead (Smith et al., 2008).

Another key element for determining the signal-to-noise ratio (SNR) of the detected EPR signal is a low-noise amplifier (LNA) used directly after the resonator. It is becoming increasingly popular to improve spectrometer sensitivity (Bienfait et al., 2016; Pfenninger et al., 1995; Šimėnas et al., 2021; Kalendra et al., 2023; Jbara et al., 2025; Rinard et al., 1999). An additional LNA inserted into the probehead can help to minimize the noise contribution of the circulator or any similar transmit–receive decoupling circuit, as well as a protection switch for the LNA inside the mw bridge of the EPR spectrometers. In principle, if the LNA is used as the first device after the resonator then all other components no longer play any significant role for the SNR. However, in most cases, the compatibility of such probes with commercial EPR spectrometers becomes a non-trivial task requiring some modification of the mw bridge. In particular, a strong ring-down signal can lead to the LNA saturation or damage, while protection switches, in turn, result in additional noise. Here, we demonstrate the use of a bimodal cavity embedded in a Q-band probe, combined with an additional LNA to improve the sensitivity of a Q-band EPR spectrometer. For our test measurements, the LNA is placed outside the cryostat at room temperature to avoid complications caused by the external static magnetic field and low temperatures. The probehead can be used with commercial EPR spectrometers without any modification of the setup. The probe was tested by means of pulsed EPR with various samples at room temperature and at 80 K. The isolation between the excitation and detection mode makes it possible to take full advantage of the LNA and noticeably improve the sensitivity of the spectrometer.

## 2 Development of the probehead

**Figure 1:**
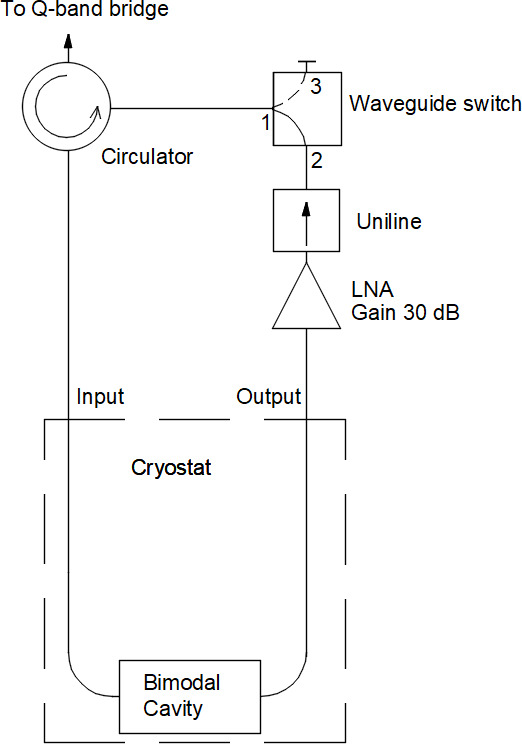
Block diagram of the Q-band EPR probehead. The waveguide switch is shown in position 1–2 for transmission-mode operation. Reflection mode is available when the switch is in position 1–3.

The block-diagram of the probehead with a cavity is shown in the Fig. 1.

The probehead is designed to operate with the Bruker ELEXYS E580 EPR spectrometer. The probehead dimensions are compatible with that of Bruker flexline Q-band resonators, and it fits perfectly into an Oxford CF935 Helium flow cryostat to be able to operate at temperatures in the range of 5–300 K. The probe can be connected to the Q-band bridge of the Bruker ELEXSYS E580 EPR spectrometer by means of a standard WR-28 waveguide.

**Figure 2:**
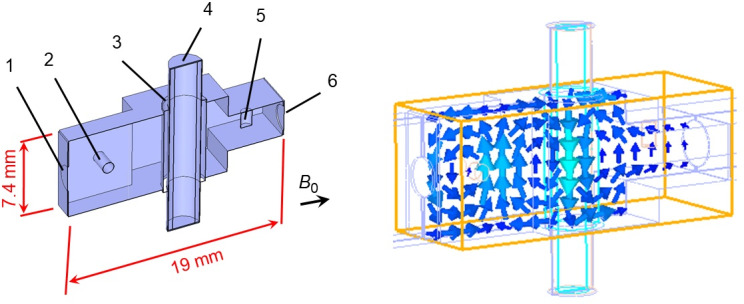
Bimodal TE103 cavity. On the left: 1 – input port with a coupling iris of 2.2 mm diameter, 2 – quartz rod of 1 mm diameter for input-mode frequency tuning, 3 – Teflon tube of 4 mm OD/3 mm ID for resonator protection against impurities, 4 – quartz sample tube of 2.8 mm OD, 5 – quartz rod of 1.5 mm diameter for output-mode frequency tuning, 6 – output port with a coupling iris of 3 mm diameter. Input and output ports have similar 7.4×3.7 mm cross-sections. All other dimensions of the structure are included in a 3D model in the Supplement. On the right: microwave $B_{1}$ field distribution along the cavity demonstrating the decoupling between output and input ports (simulated by CST Suite).

In the probe, we use a bimodal cavity based on the design described by James Hyde and coworkers, scaling it up for Q-band applications. This is a bimodal cavity (Fig. 2) in which two rectangular TE103 modes are polarization-crossed and have two half-wavelengths common (Hyde et al., 1968). The great merit of this kind of cavity is the significant isolation between the input and output modes that can be achieved with a sample volume of 20–50 µ L over a wide temperature range.

The probehead can operate in reflection or transmission mode, depending on the position of the manual waveguide switch (530B/383 MI-Wave Inc., USA). The reflection mode of operation is typical for conventional probes and will not be described further here. In transmission mode, the probehead operates in combination with an LNA. Our used LNA (Model JS-426004000-27-10P Narda-MITEQ, USA) has a 1.9 dB noise figure at 20 °C, which corresponds to an equivalent noise temperature of 160 K. For our test experiments, it is placed outside the sample cryostat. The uniline (4IWN32-2 Dorado Int., USA) protects the LNA from reflected mw power. A circulator (Model 179B-34/383 Anritzu Inc., Japan) with 0.13 dB insertion loss and 33 dB isolation at 34 GHz was chosen to direct the mw power from the Q-band bridge to the bimodal cavity and from the LNA back to the receiver part of the spectrometer.

## 3 Experimental results

**Figure 3:**
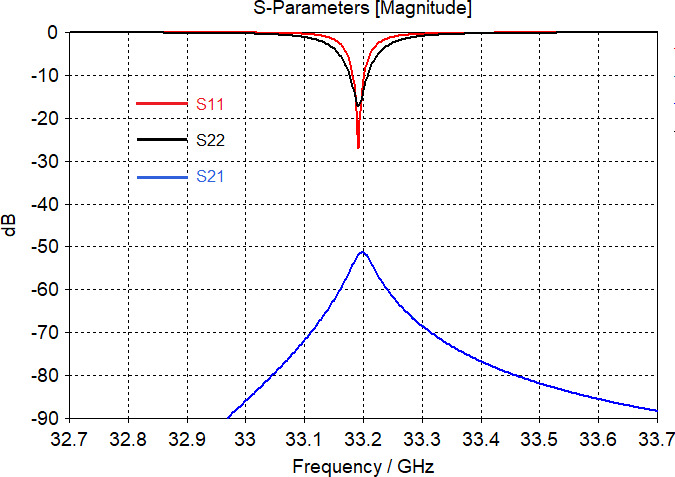
Microwave properties of TE103 bimodal cavity simulated by CST Suite: S11 (red) – input port reflection coefficient, S22 (black) – output port reflection coefficient, S21 (blue) – transmission coefficient that indicates output-to-input isolation between input and output ports.

**Figure 4:**
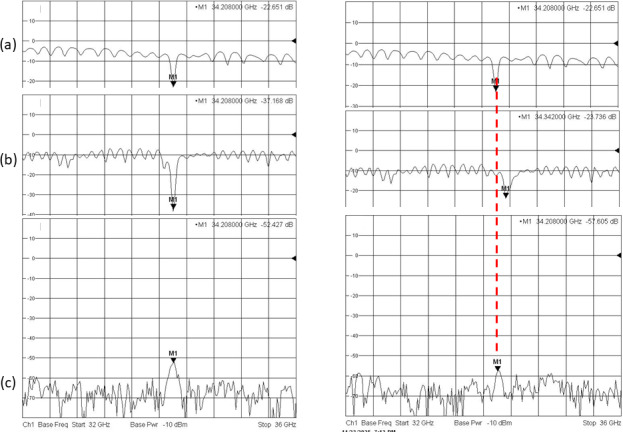
**(a)** Return loss of the probehead input for switch position 1–3. **(b)** Return loss of the cavity output when the network analyzer is connected to the output position as indicated in Fig. 1. **(c)** Input-to-output isolation of the probehead without the LNA. On the left: input and output modes are tuned to the same frequency of 34.208 GHz. On the right: output mode was detuned to 34.342 GHz (134 MHz above the input mode).

The probehead loaded with a frozen aqueous solution (with a dielectric constant of ε= 3.4 and a loss tangent of tg δ= 0.01) in a quartz sample tube has been simulated in the 33–34.5 GHz range by finite-element calculations with CST Suite version 2021. The simulation results are frequency-dependent s parameters: S11 is the reflection coefficient of the input cavity mode, S22 is the reflection coefficient of the output cavity mode, and S21 is the transmission coefficient between the input and output of the structure. Both modes of the cavity are tuned to the same frequency of 33.192 GHz. In this case, isolation between the input and output modes characterized by an S21 curve is approximately 51 dB at the frequency of interest (Fig. 3), which was reached without additional tuning paddles. The resonance frequencies of the empty cavity are higher than the spectrometer frequency range and are shifted by the sample in the quartz tube of OD = 2.8 mm, ε= 3.8 (Rototec-Spintec, USA) down into the 33–34.5 GHz range. Microwave performance of the probehead was tested using a network analyzer (ZVA-40 Rohde&Schwarz) at 294 K on a sample of BDPA:PS powder and on a sample of 0.1 mM OXO TEMPO (Sigma-Aldrich GmbH) in toluene at 80 K. The results of the experimental tests on the OXO TEMPO sample are shown in Fig. 4, with traces representing the reflection coefficient S11 of the input mode (a), the reflection coefficient S22 of the output mode (b), and the transmission coefficient S21 which characterizes the input-to-output isolation (c).

Traces (a) and (b) on the network analyzer screen are return loss traces. Their shift down to ∼ 4 and ∼ 8 dB relative to the 0 dB level is due to propagation losses in UT 141 coaxial cables with SMA connectors connecting the probe to the network analyzer and insertion losses in the mw components inside the probe. All together, mismatches in long transmission paths cause some standing waves in traces (a) and (b). Trace (c) indicates the minimum isolation at the resonant position. Logically, if the two modes resonate at the same frequency (Fig. 4, on the left) then the isolation will be lower compared to the case of any frequency offset between the two modes (Fig. 4, on the right). In the presented graph, a 134 MHz frequency offset between both modes provides higher isolation by 5 dB. This dependence of isolation on frequency offset can be used as a tool for indirectly monitoring the output mode and adjusting it to the frequency of the input mode by means of Xepr on the Bruker ELEXYS E580.

The measured decoupling between the input and output of the resonator is approximately (52 dB - (4 dB + 8 dB) /2 ) =46 dB. This experimental value is a few decibels worse than the simulated one due to the imperfections of the inner surfaces of the fabricated resonator structure and due to the presence of a sample tube which may slightly shift from the axis in the experiments. Q factors of the input and output modes of the bimodal resonator are $Q_{\mathrm{input}}=$ 250 and $Q_{\mathrm{output}}=$ 180.

**Figure 5:**
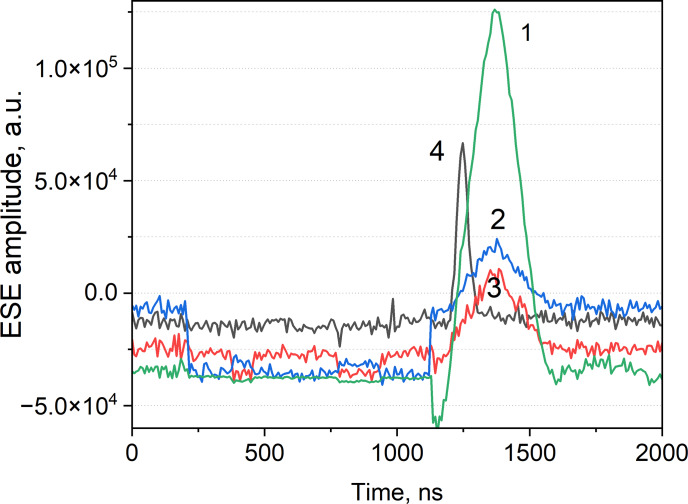
Hahn echo with BDPA:PS in a 0.5 mm ID quartz capillary measured at 294 K in (1) a bimodal probehead in transmission mode with LNA (Patt = 18 dB; π/2 -pulses = 80 ns; π -pulses = 160 ns), (2) a bimodal probehead without LNA in reflection mode, (3) an EN 5107D2 probehead (Patt = 24 dB; π/2 -pulses = 80 ns; π -pulses = 160 ns), and (4) an EN 5107D2 probehead (Patt = 0 dB; π/2 -pulses = 6 ns; π -pulses = 12 ns). All traces were recorded with a single shot per point and with 400 ns delay between the pulses.

The assembled probehead was tested using electron spin echo (ESE) experiments on a Bruker ELEXYS E580 EPR spectrometer equipped with a 150 W traveling-wave-tube (TWT) amplifier (Applied Systems Engineering Inc., USA). For testing at room temperature, we use BDPA:PS powder with a total of $10^{15}$ spins in a 0.5 mm ID fused quartz capillary (VitroCom, USA). The sample capillary was placed in the probehead with a 2.8 mm OD sample tube and measured in both reflection and transmission modes when both modes were set to the same frequency. The same sample in the same capillary was also measured with a Bruker EN 5107D2 probehead. The echo signals of both probeheads (bimodal and Bruker EN 5107D2), normalized to the same noise level, are presented in Fig. 5.

In the case of the bimodal probehead, the mw attenuation was set to 18 dB in order to avoid overload and damage of the LNA that resulted in optimal π/2 -pulses = 80 ns; π -pulses = 160 ns pulses. A similar pulse sequence was used with the EN 5107D2 probehead, setting mw attenuation to 24 dB. This test showed a 4-fold improvement in the signal-to-noise ratio (SNR) for the bimodal probehead equipped with the LNA. For comparison, full mw power (0 dB attenuation) was also applied to the EN 5107D2 probehead with optimal π/2 -pulses = 6 ns; π -pulses = 12 ns pulses. In this case the SNR improvement using the bimodal probe is still achieved 2 times over by measuring the peak amplitudes of the echo signals. For inhomogeneously broadened spectra, the echo width follows the excitation pulse width in time domain traces. That is the reason that the echoes excited by short (12 ns) pulses look much shorter in comparison with the echoes excited by 160 ns pulses (Fig. 5). In the case of longer pulses, the burned hole in the EPR spectrum is narrower, causing a smaller number of excited spins with respect to the case of shorter excitation pulses, resulting in a difference in terms of the signal intensities between trace 3 and trace 4 that is usually measured by integrated echoes. In the case of excitation by pulses of similar pulse length, the echoes can also be compared in terms of peak intensities because this is the simplest and most accurate approach to show the better performance of the bimodal probe head with LNA (trace 1) with respect to its operation without LNA (trace 2), as well as the performance of the Bruker probe (trace 3). However, in comparison with shorter pulses (trace 4), integrated echo values can be used. In this case, the better performance of the bimodal probe with LNA is also evident if integrated intensities instead of peak intensities for traces 1 and 4 will be used. Q factors of the input and output modes of the bimodal resonator have been chosen to be low enough ( $Q_{\mathrm{input}}=$ 250 and $Q_{\mathrm{output}}=$ 180) to reach a broad EPR excitation.

**Figure 6:**
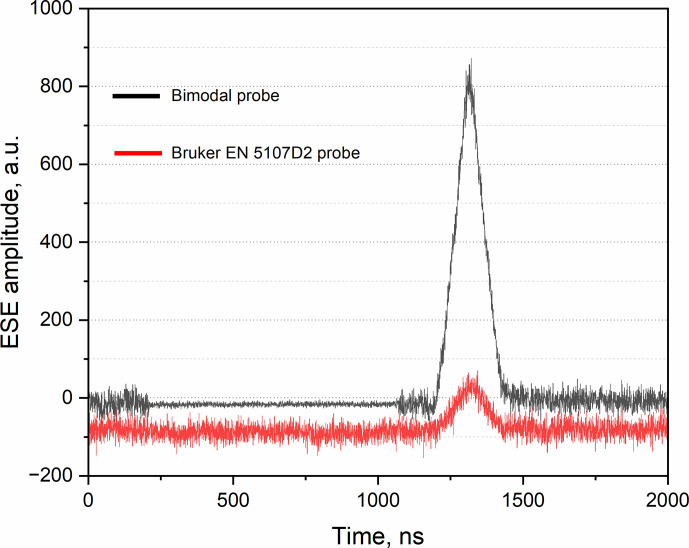
Spin echo with 0.1 mM OXO TEMPO in toluene measured at 80 K by a typical Hahn echo experiment in (black) a 2.8 mm OD sample tube inside the bimodal probehead (Patt = 15 dB; π/2 -pulses = 50 ns; π -pulses = 100 ns) and (red) a 1.6 mm OD sample tube inside the EN 5107D2 probehead (Patt = 22 dB; π/2 -pulses = 50 ns; π -pulses = 100 ns). All traces were recorded with a single shot per point and with 400 ns delay between the pulses. Video gain is 6 dB. In the region between 200 ns and 1100 ns where the spectrometer protection gate is ON, the noise for the bimodal cavity trace (the black one) is lower because it is reduced proportionally due to the normalization step of the large signal produced by the LNA, which has 30 dB gain.

We also accomplished another Hahn echo experiment using a 0.1 mM TEMPO in a toluene sample in 2.8 mm OD and 1.6 mm OD sample tubes for the bimodal and Bruker EN 5107D2 probeheads, respectively. This experiment was performed at 80 K. The experimental results of this test are shown in Fig. 6.

In this case, the signal-to-noise enhancement reaches a factor of 7 but only with a lower mw power and longer pulse lengths. The difference in terms of the results of this test and the previous one is mainly due to a larger number of spins in the 2.8 mm OD sample tube used in the bimodal probehead with respect to the 1.6 mm OD sample tube used in the Bruker probehead, resulting in sample volumes of 20 and 6 µ L, correspondingly. Thus, the 3.5-times enhancement is achieved due to a larger sample volume in the bimodal probe, and the additional 2-times SNR improvement is due to the LNA application. It should be noted that the reliability of this comparison depends on the specific performance of the commercial EN 5107D2 probe. Our EN 5107D2 probehead is not a new one and may have a slightly lowered mw power conversion factor compared to other (new) commercial probes. All presented experiments have been accomplished with the available spectrometer hardware and software.

The obtained SNR enhancements (peak amplitude values) are summarized in the Table 1.

**Table 1:** Signal-to-noise enhancements for the bimodal probehead with respect to the EN 5107D2.

Sample	Pulse width, ns	SNR enhancement,	Remarks
		times	
BDPA:PS at 294 K	80/160	4	
	6/12	2	Due to mw power limitations in bimodal probe
OXO TEMPO at 80 K	50/100	7	3.5 times – due to difference in sample volumes
			and 2 times – due to the additional LNA

## 4 Discussion

The aim of this study is to act as a proof-of-principle demonstration of the improvements obtained by using a bimodal resonator combined with an LNA in the detection channel directly after the resonator. There is still the prerequisite to use reduced mw pulse power for the spin excitation due to the limited isolation of 46 dB between the input and output of the mw resonator. This means that, at the moment, it is not possible to use the full 150 W power of the traveling-wave tube amplifier. However, despite the restrictions caused by the modest input-to-output isolation of our newly developed probehead, we were able to demonstrate a significant SNR enhancement compared to the standard reflection mode operation of the probe, as well as with a commercial Q-band probehead. The developed probehead may be interesting for use in time-resolved EPR methods (Biskup, 2019) that do not require such high mw power. Such methods include transient EPR (Niklas and Poluektov, 2017; Tait et al., 2015) and non-adiabatic rapid-scan EPR (NARS) (Kittell et al., 2011; Stoner et al., 2004; Rokeakh and Artyomov, 2023). In addition, for pulse EPR applications with broadband mw pulses, the bimodal resonator might offer a significant advantage in avoiding standing waves compared to resonators in the reflection mode (Trenkler et al., 2025).

The isolation level can be further improved by introducing tune paddles into the resonator (Mailer et al., 1980), which we plan to do in the future to extend the probe to high mw power. Another improvement will be a cryogenic LNA which is placed into the cryostat to reduce the noise temperature at the input of the LNA from 160 K to a supposed 10–50 K (Kalendra et al., 2023), as well as to further reduce the noise figure of the LNA itself. However, the presence of strong magnetic fields can impair the operation of the LNA due to the Hall effect (Harrysson Rodrigues et al., 2019), which should be eliminated by proper shielding from the magnetic field or by careful orientation of the LNA. In addition, the problem associated with repeated cooling and warming cycles of the probe can lead to a shorter LNA lifetime.

## 5 Conclusions

This work showed that a bimodal cavity in combination with an LNA connected to the mw resonator in transmission mode led to an improvement in SNR in pulse EPR experiments performed at Q-band frequencies. For simplicity, the LNA is placed outside the cryostat at room temperature, which provides a noise figure of 1.9 dB at 34 GHz. As a result, we achieved an experimental SNR enhancement factor of 2 to 4 regardless of the sample temperature and composition of the sample. Another feature of the proposed probehead is its compatibility with commercial Bruker ELEXYS EPR spectrometers without any modification of the mw bridge.

## Supplement

10.5194/mr-7-21-2026-supplement The Supplement contains the following file: bimodal cavity simulation TE103_induction.cst, which was used to optimize the geometry of the structure. The supplement related to this article is available online at https://doi.org/10.5194/mr-7-21-2026-supplement.

## Data Availability

The experimental data are available at the Goethe University Data Repository under 10.25716/gude.14gh-qa3n (Denysenkov, 2026).
