# Supplementary material for: Bimodal Q-band probehead with improved signal-to-noise ratio in pulse electron paramagnetic resonance
Source: Magn Reson (Gott). 2026 Mar 20;7(1):21–8. doi: 10.5194/mr-7-21-2026 (PMC13055999; doi:10.5194/mr-7-21-2026)
Supplement: The Supplement contains the following file: bimodal cavity simulation TE103_induction.cst, which was used to optimize the geometry of the structure. The supplement related to this article is available online at https://doi.org/10.5194/mr-7-21-2026-supplement. [file mr-7-21-2026-supplement.zip › mr-7-21-2026-supplement-title-page.pdf]

Supplement of Magn. Reson., 7, 21–28, 2026
https://doi.org/10.5194/mr-7-21-2026-supplement
© Author(s) 2026. CC BY 4.0 License.

Open Access
MAGNETIC
RESONANCE

Supplement of

Bimodal Q-band probehead with improved signal-to-noise ratio in pulse electron paramagnetic resonance

Vasyl Denysenkov et al.

Correspondence to: Vasyl Denysenkov (denysenkov@em.uni-frankfurt.de)

- mr-7-21-2026-supplement-title-page.pdf
- TE103 bimodal for paper.cst

The copyright of individual parts of the supplement might differ from the article licence.
